# Uremic Toxins and Cardiovascular Risk in Chronic Kidney Disease: What Have We Learned Recently beyond the Past Findings?

**DOI:** 10.3390/toxins14040280

**Published:** 2022-04-14

**Authors:** Carolla El Chamieh, Sophie Liabeuf, Ziad Massy

**Affiliations:** 1 Center for Research in Epidemiology and Population Health (CESP), Paris-Saclay University, Versailles-Saint-Quentin-en-Yvelines University (UVSQ), INSERM UMRS 1018, F-94807 Villejuif, France; carolla.el-chamieh@inserm.fr; 2 Pharmacology Department, Amiens University Hospital, F-80000 Amiens, France; 3 MP3CV Laboratory, EA7517, Jules Verne University of Picardie, F-80000 Amiens, France; 4 Nephrology Department, Ambroise Paré University Hospital, APHP, F-92100 Paris, France

**Keywords:** uremic toxins, atheromatous cardiovascular diseases, non-atheromatous cardiovascular diseases, chronic kidney disease

## Abstract

Patients with chronic kidney disease (CKD) have an elevated prevalence of atheromatous (ATH) and/or non-atheromatous (non-ATH) cardiovascular disease (CVD) due to an array of CKD-related risk factors, such as uremic toxins (UTs). Indeed, UTs have a major role in the emergence of a spectrum of CVDs, which constitute the leading cause of death in patients with end-stage renal disease. The European Uremic Toxin Work Group has identified over 100 UTs, more than 25 of which are dietary or gut-derived. Even though relationships between UTs and CVDs have been described in the literature, there are few reviews on the involvement of the most toxic compounds and the corresponding physiopathologic mechanisms. Here, we review the scientific literature on the dietary and gut-derived UTs with the greatest toxicity in vitro and in vivo. A better understanding of these toxins’ roles in the elevated prevalence of CVDs among CKD patients might facilitate the development of targeted treatments. Hence, we review (i) ATH and non-ATH CVDs and the respective levels of risk in patients with CKD and (ii) the mechanisms that underlie the influence of dietary and gut-derived UTs on CVDs.

## 1. Introduction

With an estimated prevalence between 8% and 16%, chronic kidney disease (CKD) is a growing worldwide public health problem [1]. The Kidney Disease: Improving Global Outcomes (KDIGO) initiative has defined CKD as a decrease in kidney function for at least 3 months, referring a glomerular filtration rate (GFR) below 60 mL/min or an albumin-to-creatinine ratio above 30 mg/g (3.4 mg/mmol) [2]. Renal impairment has been directly linked to high rates of morbidity and mortality in general [3] and cardiovascular morbidity and mortality in particular [4,5]. In fact, CKD increases the risk of cardiovascular disease (CVD) by a factor of two to four [6], making them the leading cause of death in long-standing CKD patients [4]. In adults, CKD is associated with both atheromatous (ATH) CVDs (such as myocardial infarction (MI) and stroke) and non-atheromatous (non-ATH) CVDs (such as heart failure and atrial fibrillation) [7].

This increased risk of CVD is multifactorial given the combination of both, traditional cardiovascular risk factors that are often found as comorbidities in patients with CKD (e.g., hypertension, diabetes mellitus (DM), dyslipidemia, advanced age, male sex, smoking, high body mass index) and non-traditional cardiovascular risk factors, known as CKD-related risk factors (such as high albuminuria and uremic toxins’ (UTs) accumulation) [8,9].

UTs are defined by harmful solutes accumulated in the body when the kidneys’ filtration capabilities are gradually lost, whereas they normally should be excreted by healthy kidneys [4,10,11]. They originate from endogenous metabolism, microbial metabolism, or exogenous intake. In 2021, the European Uremic Toxin Work Group (EUTox) identified over 100 UTs, including more than 25 dietary and gut-derived compounds [12,13]. Based on their molecular weight and protein-binding ability, UTs are commonly classified into three categories: free water-soluble, low-molecular-weight solutes (<500 Da), water-soluble middle molecules (>500 Da), and protein-bound solutes [12,14]. In December 2021, Rosner et al. suggested a novel six-category classification of UTs [11]: small protein-bound molecules (<500 Da), small water-soluble molecules (<500 Da), small-middle molecules (500–15,000 Da), medium-middle molecules (>15,000–25,000 Da), large-middle molecules (>25,000–58,000 Da), and large molecules (>58,000 Da). This more holistic classification included the UTs’ physicochemical characteristics and their correlations with clinical symptoms and outcomes; however, Rosner et al.‘s classification has some limitations and has not yet been validated [11].

**Figure 1:**
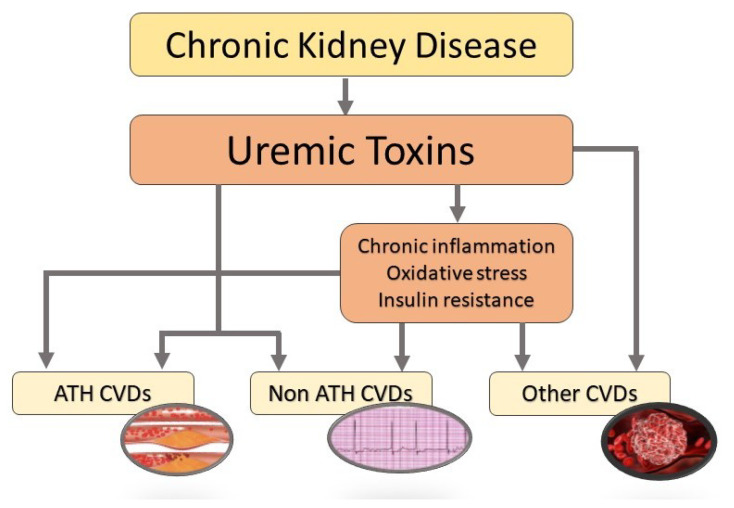
Schematic description of the effects of uremic toxins on cardiovascular diseases in a CKD setting. ATH, atheromatous; CVDs, cardiovascular diseases.

Dietary metabolites (such as 3-carboxy-4-ethyl-5-propyl-2-furanpropanoic acid (CMPF), phosphate, and urea) are known to be strongly associated with cardiovascular events [15,16]. Phosphate’s impact on CVDs does not occur independently: Phosphate has a direct effect on vascular smooth muscle cells (VSMCs) through the complex calcium/phosphate and an indirect effect via the fibroblast growth factor 23 (FGF23)/Klotho and PTH axes [17]. The gut microbiota metabolizes many dietary compounds, although most of the resulting metabolites are excreted by the kidneys in healthy individuals [18]. Subsequently, any disruption of the intestinal microbial composition, as seen in patients with CKD, will generate harmful metabolites [13]. Here, we only review data on the UTs with the greatest toxicity in vitro and in vivo [16]: indoles (indoxyl sulfate (IS), indole-3-acetic acid (IAA), kynurenine, and kynurenic acid (KA)), phenols (p-cresyl sulfate (PCS), p-cresyl glucuronide (PCG), and phenylacetylglutamine (PAG)), hippurates (hippuric acid (HA)), and others (CMPF, phosphate, urea, trimethylamine N-oxide (TMAO)). As ilustrated in Figure 1, the accumulation of these UTs has been implicated directly or indirectly in a spectrum of ATH and non-ATH CVDs [19].

Previous reviews described the endogenously generated UTs (such as β-2 microglobulin, interleukins, and other inflammatory markers), we aimed in the present narrative review to focus on the most toxic dietary and gut-derived compounds. Here, we review research published between 2002 and 2022 by (i) defining the various ATH and non-ATH CVDs and the associated risks in patients with CKD (ii) and listing the various mechanisms that underlie the influence of dietary and gut-derived UTs on the CVD risk.

## 2. Cardiovascular Diseases

The elevated incidence and prevalence of CVD is due to many conventional and non-conventional risk factors, of which CKD is the major non-modifiable one [20]. The prevalence of CVD is higher in patients with CKD than in healthy individuals, and the cardiovascular mortality rate is 10 to 30 times higher in dialysis patients than in the general population [21]. This elevated risk can be ascribed to the combination of conventional cardiovascular risk factors with those related directly to CKD such as oxidative stress, chronic inflammation, vascular calcification, and UTs [8,20,22]. According to Jankowski et al. [22], “CKD mimics accelerated aging of the cardiovascular system”.

In physiopathologic terms, CVDs can be classified as ATH or non-ATH [6] that are both highly prevalent in patients with CKD [8].

### 2.1. Atheromatous Cardiovascular Diseases

ATH CVD is characterized by the presence of occlusive lesions and plaques (called atheromas) inside the arterial wall; the aorta and the coronary arteries are primarily affected [23]. Atheromas in patients with CKD are mainly characterized by important thickening of the intima media [20,24]. According to the KDIGO [25] and the Cardiovascular Stroke Endpoint Definitions for Clinical Trials [26], ATH CVDs include stroke or MI (whether fatal or not) and hospitalization for silent ischemia, unstable angina, transient ischemic attacks, intrastent thrombosis, peripheral artery disease (PAD), percutaneous coronary interventions or coronary artery bypass grafts, vascular surgery, amputation, and revascularization for coronary artery disease (CAD) or PAD. Furthermore, PAD can be defined as a history of amputation, angioplasty, or lower limb bypass prompted by ATH distal ischemic lesions [8,27].

The prevalence of atherosclerotic CVD is higher in patients with moderate CKD than in healthy individuals, and atherosclerosis progression is strongly linked to the worsening of CKD [28]. The incidence of fatal MI was higher in dialysis patients than in the general population [20]. The results of observational studies have shown that the stroke risk increases with declining GFR [28,29], and that CKD is associated with a higher risk of PAD [30,31].

### 2.2. Non-Atheromatous Cardiovascular Diseases

Non-ATH CVD encompasses other types of CVD, including sudden cardiac death or death from heart failure (without a history of CAD), hospitalization for heart failure (again with no history of CAD), cardiac fibrosis, atrial fibrillation or other arrhythmia disorders, diastolic dysfunction, arterial stiffness, cardiomyocytes hypertrophy, vascular calcification, or valvular heart disease [8,27,32]. Prior cohorts concluded that the prevalence of heart failure increased markedly with CKD progression and affected 65 to 70% of patients with end-stage renal disease (ESRD) [33]. Patients with CKD had a significantly higher aortic pulse wave velocity [34]. The higher the CKD stage, the greater the risk of arterial stiffness [35]—the most prevalent arterial modification being in patients with CKD [36]. Arterial stiffness reflects vascular calcification [37], and both variables are independent predictors of CVD mortality [38]. Vascular calcification is mainly associated with low vessel elasticity [39], and medial artery calcification is the most common vascular calcification in patients with CKD [38]. Various studies have reported that CKD is a risk factor for cardiac arrhythmia [40,41]. Although atrial fibrillation represents the most prevalent type of arrhythmia, ventricular disorders are the most lethal [40,42]. For example, ventricular tachyarrhythmia accounted for 79% of cardiac arrests recorded in a study of hemodialysis (HD) patients [42].

In summary, CKD contributes significantly to severe non-ATH CVDs [33].

### 2.3. Other than Atheromatous and Non Atheromatous Cardiovascular Diseases

We also included cardiovascular complications that are neither ATH CVDs nor non-ATH CVDs (referred to hereafter as “other than ATH and non-ATH CVDs”). This group includes platelet aggregation, thrombus formation, and endothelial cell (EC) dysfunction. Many studies have characterized the vascular damage caused by CKD. It is well known that CKD progression is closely associated with levels of oxidative stress and inflammation, which enhance cardiovascular damage and mortality [43]. Numerous studies have also demonstrated that CKD is associated with significantly increased platelet activation [44,45], and with the risk of both venous and arterial thrombosis [46,47]. Patients with CKD frequently undergo endovascular procedures; the risk of post-angioplasty thrombosis or vascular access thrombosis is elevated in this high-risk population [48], and makes CKD the second-ranked risk factor for post-procedural complications [48,49].

## 3. Uremic Toxins and Risk for Cardiovascular Diseases

Some UTs markedly increase the relative risk of CVD in the CKD population. Many in vitro, in vivo, and observational studies have concluded for the impact of dietary and gut-derived UTs on adverse cardiac risk. We systematically searched the literature (the PubMed database, up until 28 February 2022) for publications on the relationship between UTs and CVDs in patients with CKD, by combining the following keywords: (“Chronic kidney disease” OR “renal impairment” OR “kidney disease” OR “chronic kidney failure” OR “chronic renal failure”) AND (“Cardiovascular disease” OR “vascular calcification” OR “oxidative stress” OR “coronary artery disease” OR “atherosclerosis” OR “peripheral artery disease” OR “cardiovascular damage” OR “Cardiovascular Diseases” [Mesh] OR “arrythmia” OR “stroke” OR “cardiovascular death” OR “myocardial infarction”) AND (“uremic toxin*” OR “uremic retention solute*” OR “cardiovascular toxin*” OR “ indoxyl sulfate” OR “p-Cresyl sulfate” OR “p-cresyl glucuronide ” OR “indole-3-acetic acid “OR” hippuric acid” OR “kynurenic acid” OR “kynurenine” OR “3-carboxy-4-methyl-5-propyl-2-furanpropanoic acid” OR “Phenylacetylglutamine” OR “trimethylamine N-oxide”) AND (“in vivo” OR “in vitro” OR “animal*”) NOT “hemodialysis” NOT “peritoneal dialysis” NOT “dialysis”). A total of 262 articles were analyzed. We excluded 9 studies on pediatric patients, 25 reviews, and 15 studies investigating other UTs than the dietary and gut-derived ones included in the present review. A total of 213 original articles were selected.

### 3.1. Experimental Data: In Vitro Studies

Table 1 summarizes the in vitro studies of the UTs’ impact on CVDs in a CKD setting.

Recent results have demonstrated that UTs contribute to CVDs in CKD patients through a variety of mechanisms, the most significant of which are endothelial dysfunction and vascular calcification caused mostly by oxidative stress and inflammation. For example, a recent study of primary human dermal microvascular ECs by Arinze et al. [50] showed that tryptophan-derived indoles (IS, kynurenine, and KA) were associated with the worsening of PAD. The UTs decreased Wnt/β-catenin activity, causing EC dysfunction and impaired angiogenesis.

Studies in different in vitro models found that IS, IAA, PCS, phosphate, and urea induced oxidative stress by increasing reactive oxygen species (ROS) production [51,52,53,54,55,56,57] or by activating leukocyte free radical production [58], thus contributing to endothelial dysfunction and apoptosis. Itoh et al. [59] reported that IS induced ROS production in human umbilical vein endothelial cells (HUVECs) more intensely than CMPF did. HA and phosphate caused EC apoptosis by disrupting the mitochondrial membrane through increased production of ROS and Drp1 protein [54,60].

Shang et al. [61] concluded that IS, IAA, and HA increased levels of miR-92a, a microRNA induced by oxidative stress in ECs and which is involved in atherosclerosis. Furthermore, IS was involved in the pathophysiology of atherosclerosis by increasing the expression of the adhesion molecules intercellular adhesion molecule-1, vascular cell adhesion molecule-1, monocyte chemotactic protein-1, and e-selectin [62,63], and activating the p42/44 mitogen-activated protein kinase (MAPK) pathway and thus vascular smooth muscle cell (VSMC) proliferation [64].

Experiments on HUVECs demonstrated that PCS had a damaging effect on ECs by (i) directly stimulating the Rho-associated protein kinase [65], (ii) enhancing NADPH oxidase expression and ROS production [53], (iii) inducing the shedding of endothelial microparticles [66], and (iv) inhibiting (along with IS) endothelial proliferation and wound repair [67]. Phosphate (the UT most intensively studied in vitro) also damages ECs. Phosphate overloads blocked G1/S progression, reducing EC proliferation [68]. Moreover, phosphate overload decreased annexin II expression and stiffened ECs [69]. In experiments on aortic rings, excess of phosphate caused vasoconstriction, increased phenylephrine-induced contraction, and lowered acetylcholine-induced relaxation [70]. Furthermore, high phosphate levels accelerated calcium deposition on arteriole walls and decreased vasorelaxation and nitric oxide (NO) production in human vascular smooth muscle cells (HVSMCs), leading to vasoconstriction [71]. Similarly, urea disrupted ECs either via a direct effect on the cells or indirectly via protein carbamylation. Urea prompted the formation of excessive neutrophil extracellular trap in HUVECs [72], altered cell-to-cell junctions in an immortalized human EC line [73], induced Associated Agonist Of Cell Death (BAD) protein expression [74], and inhibited glyceraldehyde 3-phosphate dehydrogenase (GAPDH) and Prostacyclin (PGI2) synthase (thus facilitating the activation of pro-atherosclerotic pathways) [57]. It is also noteworthy that high urea levels were associated with elevated mitochondrial ROS production in arterial ECs even after dialysis—suggesting that there is a “cellular memory” for urea-induced oxidative stress [55]. In terms of an indirect effect, urea levels were positively correlated with high-density lipoprotein (HDL) carbamylation, which then inhibited endothelial repair functions [75]. TMAO had the same endothelial effect in various models. It activated the nucleotide-binding domain, leucine-rich-containing family, pyrin domain-containing-3 (NLRP3) inflammasome and nuclear factor-kappa B (NF-κB) signals, promoted leukocyte-EC adhesion, EC dysfunction, and vascular calcification and thus helped to enhance atherosclerosis processes [76,77,78,79].

Vascular calcification happens in various ways. Studies of human aortic smooth muscle cells (HASMCs) showed that IS induced aortic calcification by activating the NF-κB signaling pathway [80], decreasing Klotho expression [81], and inducing ROS generation and the expression of NADPH oxidases (Nox1, Nox2, and Nox4), core binding factor 1, alkaline phosphatase, and osteopontin [51]. In Bouabdallah et al.’s study [82] of HUVECs and HASMCs, IS and phosphate induced the secretion of interleukin-8 from ECs and thus the promotion of vascular calcification. In addition, phosphate promoted extracellular matrix calcification and upregulated osteoblast marker expression by VSMCs [83]. High phosphate levels activated toll-like receptor 4 (TLR4)/NF-κB signaling [84] and upregulated aldosterone synthase expression, which induced VSMCs osteogenic transdifferentiation and calcification [85]. In an in vitro study of peripheral blood mononuclear cells, phosphate modulated miR-223 expression and decreased osteoclastogenesis [86]. Similarly, phosphate caused osteoblastic differentiation in VSMCs [87], reduced perlecan expression in rat aortic rings (ex vivo) and HVSMCs, and induced BMP-2 (involved in osteogenic transdifferentiation pathways) [88]. Lastly, phosphate mediated vascular calcification by increasing alkaline phosphatase activity in VSMCs [89].

It has been reported that IS contributes to CVD through associations with arrhythmia, cardiac hypertrophy, and fibrosis. The toxin’s arrhythmogenic effect was evaluated in embryonic rat cardiomyocytes: IS inhibited the inward rectifier potassium ion channel—prolonging the action potential and the QT interval and inducing early after depolarization [90]. Lekawanvijit et al. [91] demonstrated the fibrotic effect of IS for the first time; this was later confirmed by Liu et al. [92]. IS exerted pro-fibrotic, pro-hypertrophic, and pro-inflammatory effects by activating MAPK and NF-κB pathways [91]. Furthermore, IS increased collagen synthesis in neonatal rat cardiac fibroblasts, promoted myocyte hypertrophy, and stimulated tumor necrosis factor-alpha and interleukin-6 expression [91]. In rats, cardiomyocytes took up IS the organic anion transporters (OATs) 1 and 3, which led to the activation of the NF-κB and MAPK pathways and favored cardiac hypertrophy and fibrosis [92].

Indoles are also known to have a prothrombotic effect. IS and IAA activated the aryl hydrocarbon receptor (AHR), which led to an increase in tissue factor (TF) expression [93,94,95]. Gao et al. [96] demonstrated that IS and IAA caused red blood cell damage, which might lead to thrombus formation.

UTs also have indirect effects on CVD. In particular, CMPF inhibited insulin secretion [97], and urea increased the expression of the adipokines retinol binding protein 4 and resistin [98].

Most of the following in vitro studies added human serum albumin (HSA) to the protein-bound UT to reflect their natural protein-bound state in uremia. This addition should be considered in such a context and, most importantly, while interpreting the results in order to reflect their actual clinical effects [99].

**Table 1:** In vitro studies of the effects of UTs on cardiovascular complications.

First Author, Year	Models	UT(s) Studied	Main Findings
Arinze [50], 2022	Primary human dermal	IS	IS, kynurenine, and KA decreased Wnt/β-catenin
	microvascular ECs	Kynurenine	activity, which causes EC dysfunction and impairs
		KA	angiogenesis.
Lano [93], 2020	HUVECs	IS	IS had a prothrombotic effect by increasing TF expression in ECs and peripheral blood mononuclear cells via AHR activation.
He [80], 2019	HASMCs	IS	IS induced calcification of HASMCs via the NF-κB signaling pathway.
Chen [81], 2016	HASMCs	IS	IS decreased Klotho expression, promoting aortic calcification.
Tang [90], 2015	Embryonic rat heart-derived cardiac H9c2 cells	IS	IS has a role in arrhythmogenesis: IS inhibited the inward rectifier potassium ion channels function, resulting in a prolonged QT interval.
Chitalia [94], 2013	HVSMCs	IS	IS increased TF expression and decreased TF ubiquitination, leading to a thrombogenic milieu.
Liu [92], 2012	Neonatal cardiac myocytes and fibroblasts from Sprague–Dawley rats	IS	IS was taken up by cardiomyocytes through OAT-1 and -3, leading to activation of the NF-κB and MAPK pathways that are involved in cardiac hypertrophy and fibrosis.
Lekawanvijit [91], 2010	Isolated NCMs, NCFs and THP-1	IS	IS has a role in harmful cardiac remodeling: it has pro-fibrotic, pro-hypertrophic, and pro-inflammatory effects via the activation of MAPK and NF-κB pathways.
Tumur [62], 2010 and Ito [63], 2010	HUVECs	IS	IS increased the expression of the adhesion molecules ICAM-1, VCAM-1, MCP-1, and e-selectin, all of which are involved in the pathophysiology of atherosclerosis.
Muteliefu [51], 2009	HASMCs	IS	IS induced ROS generation and the expression of Nox4, Cbfa1, ALP, and osteopontin in VSMCs.
Yamamoto [64], 2006	VSMCs were isolated from the aortas of male Sprague–Dawley rats	IS	IS caused VSMC proliferation via activation of the p42/44 MAPK pathway, a mechanism involved in the progression of atherosclerotic lesions.
Dou [52], 2015	Cultured human endothelial cells	IAA	IAA activated the inflammatory AHR/p38MAPK/NF-κB pathway and increased the production of endothelial ROS.
Gao [96], 2015	RBC from peripheral vein	IAA	IS and IAA caused RBC damage, which is involved
	blood of eight healthy volunteers	IS	in thrombus formation.
Gondouin [95], 2013	HUVECs	IAA	IAA increased TF expression resulting in a prothrombotic effect.
Gross [65], 2015	HUVECs and HVSMCs	PCS	PCS directly stimulated the Rho-associated protein kinase, which is involved in vascular dysfunction and vascular remodeling.
Watanabe [53], 2015	HUVECs	PCS	PCS enhanced ROS production and NADPH oxidase expression.
Meijers [66], 2009	HUVECs	PCS	PCS induced shedding of endothelial microparticles, causing endothelial dysfunction.
Schepers [58], 2007	Blood from healthy donors incubated in the presence of PCS	PCS	The presence of PCS activated pro-inflammatory leukocyte free radical production.
Dou [67], 2004	HUVECs	PCS	Both PCS and IS inhibited endothelial proliferation
		IS	and wound repair.
Huang [60], 2018	Human aortic endothelial cells	HA	HA contributed to mitochondrial fission by activating mitochondrial ROS production and Drp1 protein expression.
Shang [61], 2017	HUVECs	HA	HA, IS, and IAA increased miR-92a levels, which im-
		IS	pairs EC function.
		IAA	
Nagy [97], 2017	Human islets of Langerhans from healthy donors	CMPF	CMPF inhibited insulin secretion.
Itoh [59], 2012	HUVECs	CMPF	IS induced ROS production more intensely than
		IS	CMPF did.
Bouabdallah [82], 2019	HUVECs and HASMCs	Phosphate	Phosphate and IS induced the secretion of interleuk-
		IS	in-8 from ECs, which is involved in VSMC calcification.
Jover [83], 2018	VSMCs	Phosphate	High phosphate promoted extracellular matrix calcification and upregulated osteoblast markers.
Zhang [84], 2017	HASMCs	Phosphate	High phosphate induced vascular calcification via the activation of TLR4/NF-κB signaling.
Alesutan [85], 2017	HASMCs	Phosphate	Hyperphosphatemia upregulated aldosterone synthase expression, inducing VSMCs osteogenic transdifferentiation and calcification.
Rahabi-Layachi [68], 2015	HASMCs	Phosphate	Phosphate induced apoptosis and cell cycle arrest by blocking G1/S progression, thus reducing HASMCs proliferation.
M’Baya-Moutoula [86], 2015	Peripheral blood mononuclear cells	Phosphate	Phosphate caused vascular calcification by modulating miR-223 and decreasing osteoclastogenesis.
Ciceri [87], 2015	VSMCs	Phosphate	Phosphate caused VSMC osteoblastic differentiation and led to cell calcification.
Di Marco [69], 2013	Human coronary artery ECs	Phosphate	Hyperphosphatemia decreased annexin II expression and stiffened ECs.
Six [70], 2012	HUVECs	Phosphate	Phosphate exhibited a direct vasoconstrictor effect on aortic rings, increased phenylephrine-induced contraction, and lowered acetylcholine-induced relaxation—leading to endothelial dysfunction.
Guerrero [88], 2012	Rat aortic rings and HVSMCs	Phosphate	Phosphate reduced expression of perlecan and induced BMP-2, which is involved in the osteogenic transdifferentiation pathways and would promote cells calcification.
Shroff [89], 2010	VSMCs	Phosphate	Phosphate increased alkaline phosphatase activity and mediated calcification.
Di Marco [54], 2008	HUVECs	Phosphate	Hyperphosphatemia caused EC apoptosis by increasing ROS generation and disrupting the mitochondrial membrane potential.
Shigematsu [71], 2003	HVSMCs	Phosphate	Phosphate overload accelerated calcium deposition on arteriole walls. Moreover, phosphate led to vasoconstriction, decreased vasorelaxation, decreased NO production, stimulated ROS production, and induced ECs apoptosis.
Lee [72], 2021	HUVECs	Urea	Urea led to excessive neutrophil extracellular trap formation and thus EC dysfunction.
Maciel [73], 2018	An immortalized human EC line	Urea	Urea altered cell-to-cell junctions, leading to greater endothelial damage.
D’Apolito [55], 2018	Human arterial ECs	Urea	Abnormal high urea levels had long-lasting effects on arterial cells: urea increased mitochondrial ROS production in arterial ECs even after dialysis, which typically promotes endothelial dysfunction.
D’Apolito [56], 2017	Human endothelial progenitor cell	Urea	Urea caused ROS production and accelerated endothelial progenitor cell senescence.
Sun [75], 2016	Human arterial EC	Urea	Urea levels were positively correlated with HDL carbamylation, which inhibited endothelial repair functions.
D’Apolito [57], 2015	Human aortic ECs	Urea	Urea increased mitochondrial ROS production and inhibited GAPDH, which leads to the activation of the endothelial pro-inflammatory pathway. Furthermore, urea inactivated the anti-atherosclerosis enzyme PGI2 synthase.
Trécherel [74], 2012	HASMCs	Urea	Urea induced BAD protein expression, sensitizing the HASMCs to apoptosis.
D’Apolito [98], 2010	3T3-L1 adipocytes treated with urea	Urea	Urea increased ROS levels and expression of the adipokines retinol binding protein 4 and resistin.
Zhang [76], 2020	Aortic VSMCs from male “Sprague Dawley” rats and human VSMCs	TMAO	TMAO promoted vascular calcification through activation of the NLRP3 inflammasome and NF-κB signals.
Ma [77], 2017	HUVECs	TMAO	HUVECs showed impairment in cellular proliferation, and TMAO induced NF-κB signaling pathway, increasing vascular inflammatory signals and EC dysfunction.
Boini [78], 2017	Mouse carotid artery ECs	TMAO	TMAO activated NLRP3 inflammasomes, causing endothelial dysfunction.
Sun [79], 2016	HUVECs	TMAO	TMAO activated NLRP3 inflammasomes, causing endothelial dysfunction.

Abbreviations: AHR: aryl hydrocarbon receptor; ALP: alkaline phosphatase; Cbfa1: core binding factor 1; CMPF: 3-carboxy-4-methyl-5-propyl-2-furanpropanoic acid; CVD: cardiovascular disease; Drp: dynamin-related protein; ECs: endothelial cells; eNOS: endothelial nitric oxide synthase; ENPP1: ectonucleotide pyrophosphate/phosphodiesterase 1; GAPDH: glyceraldehyde 3-phosphate dehydrogenase; HA: hippuric acid; HASMC: human aortic smooth muscle cell; HDL: high-density lipoprotein; HUVECs: human umbilical vein endothelial cells; HVSMC: human vascular smooth muscle cell; IAA: indole-3-acetic acid; ICAM-1: intercellular adhesion molecule-1; IS:indoxyl sulfate; KA: kynurenic acid; MAPK: mitogen-activated protein kinase; MCP-1: monocyte chemotactic protein-1; NADPH: nicotinamide adenine dinucleotide phosphate; NCM: neonatal rat cardiac myocyte; NCF: neonatal rat cardiac fibroblast; NF-kB: nuclear factor-kappa B; NLRP3: nucleotide-binding domain, leucine-rich containing family, pyrin domain-containing-3; NO: nitric oxide; PCS: para-cresyl sulfate; RBC: red blood cell; ROS: reactive oxygen species; TF: tissue factor; THP-1: human leukemia monocytic cell line; TLR4: tolllike receptor 4; TMAO: trimethylamine-N-oxide; UT: uremic toxin; VCAM-1: vascular cell adhesion molecule-1; VSMC: vascular smooth muscle cells.

### 3.2. Experimental Data: Animal Studies

Animal studies investigating the UTs’ impact on ATH or non-ATH CVDs are summarized in Table 2.

#### 3.2.1. Atheromatous Cardiovascular Diseases

Arinze et al. [50,100] also recently studied the effect of tryptophan-derived indoles on PAD in adenine-induced CKD and IS solute–specific C57BL/6 mouse models [50]. IS, kynurenine, and KA increased AHR activity, resulting in the suppression of Wnt/β-catenin signaling. This phenomenon led to impaired angiogenesis and caused hindlimb ischemia. This complication of PAD was also observed by Hung et al. [101], who concluded that IS caused PAD by decreasing the mobilization of endothelial progenitor cell and impairing neovascularization. TMAO was also found to engender PAD by impairing endothelium- derived, hyperpolarizing factor-type relaxation [102]. The results of in vivo studies suggest that PCS, HA, urea, and TMAO contribute to the acceleration of atherosclerosis. In a study of five of six nephrectomized apoE –/– mice, Han et al. [103] showed that PCS induced VSMC migration and proliferation and disturbed the balance between matrix metalloproteinases and tissue inhibitors of metalloproteinases within plaques. Huang et al. [60] and Shang et al. [61] added to their in vitro findings by studying HA’s role in atherosclerosis induction in animal models. HA induced oxidative stress, led to endothelial dysfunction, impaired endothelium-dependent vasodilation [60], and induced miR-92a (involved in the angiogenic process) [61]. Recent studies have found that TMAO is also involved in atherosclerosis induction. The compound activated the expression of components of the CD36/MAPK/JNK and NF-κB signaling pathways [104,105], which promoted foam cell formation [104] and vascular inflammation [105]. Moreover, Koeth et al. [106] showed that TMAO was linked to major cardiac events—mainly MI and stroke. Massy et al. confirmed the role of urea [107] and concluded that the compound aggravated atherosclerosis by promoting arterial calcification.

#### 3.2.2. Non-Atheromatous Cardiovascular Diseases

IS, PCS, phosphate, and urea also promote non-ATH CVDs in general and vascular calcification in particular. IS and PCS induced severe calcification of the aorta and peripheral arteries: IS decreased NO production and increased endothelial nitric oxide synthase (eNOS) phosphorylation [108], and both IS and PCS activated the inflammation and coagulation pathways [109]. According to Chen et al. [81], IS promoted aortic calcification by decreasing Klotho expression. Moreover, Muteliefu et al. [110] and Adijiang et al. [111,112] showed that IS accelerated wall thickening and vascular calcification through the upregulation of p16, p21, p53, prelamin A, and osteoblast-specific protein, which induced VSMC senescence. Phosphate also generated VSMCs calcification [113] and was associated with arterial medial calcification [114,115]. Phosphate overload increased the expression of Tumor Necrosis Factor alpha (TNF-α), osteochondrogenic markers [116], and aortic runt-related transcription factor 2 [117], which induced systemic inflammation and VSMC calcification. In 5/6 nephrectomized male Sprague–Dawley rats, high phosphate caused medial calcification by increasing tissue-nonspecific alkaline phosphatase activity, which induced elastin degradation and accelerated the transformation of VSMCs into osteoblast-like cells [118].

UTs contributed to cardiac fibrosis, arrhythmia, and myocardial hypertrophy. It has been suggested that IS leads to atrial fibrillation. Exposure to IS accentuated cardiac fibrosis and cardiomyocyte hypertrophy by increasing oxidative stress and decreasing anti-oxidative defenses [119,120]. Chen et al. showed that these changes [121] led to greater pulmonary vein and atrial arrhythmogenesis. High phosphate and urea levels also lead to cardiac hypertrophy [122] and fibrosis [123]. For example, it was shown that high phosphate levels result in lower Klotho levels in rodent models of CKD [123]. Moreover, high urea levels led to systemic microvascular disease with microvascular rarefaction, tissue hypoxia, and dysfunctional angiogenesis [124]. High urea also induced systemic inflammation, which was responsible for subepicardiac artery thickening [125]. In 5/6 nephrectomized mice, PCS toxicity was linked to increases in NADPH oxidase expression and ROS production; these changes contributed to cardiomyocyte apoptosis, which in turn aggravated diastolic dysfunction (with a change in the ratio between early and late left ventricular transmitral peak flow velocities) [126].

#### 3.2.3. Other than Atheromatous and Non-Atheromatous Cardiovascular Diseases

It has been suggested that IS, kynurenine, and TMAO increase the thrombosis risk. IS activated ROS/p38 MAPK signaling and reduced Klotho expression, which would aggravate the effect on platelet aggregation and thrombus formation [127]. Kynurenine promoted clotting as a consequence of vascular injury [128]. Zhu et al. [129] concluded that TMAO enhanced submaximal stimulus-dependent platelet activation and thus contributed to the thrombosis risk.

A number of mechanisms favor EC dysfunction. In 5/6 nephrectomized rats, urea increased ROS production and thus induced oxidative stress in the systemic circulation [98,130,131]. Furthermore, high TMAO levels are associated with a decrease in NO production [132].

The oxidative stress induced by urea contributed also to insulin resistance. Urea increases oxidative stress and protein O-GlcNAcylation, thus impairing insulin secretion and glycolysis [130]. D’Apolito et al. [98] showed that urea increased ROS production and promoted insulin resistance and glucose intolerance. IS and PCS also exert indirect effects given that they were strongly associated with impaired glucose homeostasis and thus hyperglycemia and insulin resistance [109]. Koppe et al. [133] concluded that only PCS (but not PCG) induced insulin resistance by activating extracellular signal-regulated kinases and thus altering insulin signaling in skeletal muscle [134]. Furthermore, Nagy et al.’s in vitro results [97] on CMPF’s role in promoting insulin resistance were confirmed in vivo [97].

**Table 2:** Animal studies of the effects of UTs on cardiovascular complications.

First Author, Year	Models	UT(s) Studied	Main Findings
			**Atheromatous CVDs**
Arinze [50], 2022	Adenine-induced	IS	IS, kynurenine, and KA suppressed Wnt/β-
	CKD mice and IS so-	Kynurenine	catenin signaling through increased AHR activity,
	lute-specific C57BL/6	KA	leading to impaired angiogenesis and hindlimb
	mice		ischemia.
Hung [101], 2016	Mice with subtotal nephrectomy	IS	IS decreased endothelial progenitor cells mobilization and impaired neovascularization, leading to PAD.
Han [103], 2016	5/6 nephrectomized ApoE –/– mice	PCS	PCS promoted the formation of atherosclerotic lesions, induced plaque instability and the migration and proliferation of VSMCs, and disturbed the balance between matrix metalloproteinases and tissue inhibitor of metalloproteinases within the plaques.
Huang [60], 2018	5/6 nephrectomized rat model	HA	HA caused pro-atherogenic effects by contributing to endothelial dysfunction via greater oxidative stress and impaired endothelium-dependent vasodilation.
Shang [61], 2017	Male Wistar rats	HA	HA induced miR-92a, which is involved in angiogenic and atherosclerotic processes.
Massy [107], 2005	ApoE −/− mice with partial kidney ablation	Urea	Urea contributed to arterial calcification and aggravated atherosclerosis.
Matsumoto [102], 2020	Superior mesenteric arteries and femoral arteries of rat	TMAO	TMAO impaired endothelium-derived hyperpolarizing factor-type relaxation, which led to PAD.
Geng [104], 2018	Apoe −/− mice fed a high-fat diet with or without TMAO	TMAO	TMAO enhanced the expression of CD36/MAPK/JNK pathway, promoting foam cells formation and, ultimately, atherosclerosis.
Seldin [105], 2016	Female low-density lipoprotein receptor knockout mice injected with vehicle or TMAO	TMAO	TMAO induced vascular inflammation by activating MAPK and NF-κB signaling, thus enhancing atherosclerosis.
Koeth [106], 2013	Mice supplemented with dietary TMAO, carnitine, or choline	TMAO	TMAO accelerated atherosclerosis and was linked to major cardiac events.
			**Non-atheromatous CVDs**
Kuo [108], 2020	Nephrectomized male C57BL/6 mice	IS	IS promoted calcification in the aorta and peripheral arteries, with low NO production and high eNOS phosphorylation.
Opdebeeck [109], 2019	42 male Wistar rats ex-	IS	Both IS and PCS directly promoted severe calcifica-
	posed to adenine sulfate for 10 days and then fed a phosphate-enriched diet	PCS	tion in the aorta and peripheral vessels via activation of inflammation and coagulation pathways. These changes were strongly associated with impaired glucose homeostasis.
Chen [81], 2016	5/6 nephrectomized Sprague Dawley rats treated with IS	IS	IS decreased Klotho expression and promoted aortic calcification.
Chen [121], 2015	Isolated rabbit left atrium, right atrium, pulmonary vein, and sinoatrial nodes before and after treatment with IS	IS	IS may contribute to atrial fibrillation: It increased pulmonary vein and atrial arrhythmogenesis through oxidative stress, inflammation, and fibrosis.
Yisireyili [119], 2013 and Lekawanvijit [120], 2012	Dahl salt-sensitive hypertensive rats	IS	IS aggravated cardiac fibrosis and cardiomyocyte hypertrophy, with greater levels of oxidative stress and lower anti-oxidative defenses.
Muteliefu [110], 2012	Aorta of subtotally nephrectomized Dahl salt-sensitive hypertensive rats	IS	IS accelerated VSMC senescence and vascular calcification, with upregulation of p21, p53, and prelamin A through oxidative stress.
Adijiang [111], 2010	Dahl salt-sensitive hypertensive rats	IS	IS increased aortic calcification and wall thickness; induced expression of p16, p21, p53 and Rb in the calcification area; and thus promoted cell senescence.
Adijiang [112], 2008	Dahl salt-sensitive hypertensive rats	IS	IS induced aortic calcification (with expression of osteoblast-specific proteins) and aortic wall thickening.
Han [126], 2015	5/6 nephrectomized mice	PCS	PCS promoted cardiac apoptosis and diastolic dysfunction by upregulating the expression of NADPH oxidase and the production of ROS.
Hu [123], 2015	Two CKD rodent models: UNX-IRI26 and 5/6 nephrectomized	Phosphate	High phosphate was associated with lower Klotho levels, leading to cardiac hypertrophy and fibrosis.
Yamada [116], 2014	Adenine-induced CKD male Sprague–Dawley rats	Phosphate	High phosphate directly increased the expression of TNF-α and osteochondrogenic markers, inducing systemic inflammation and vascular calcification.
Lau [114], 2013	DBA/2 mice with partial renal ablation	Phosphate	High phosphate was associated with arterial medial calcification.
Crouthamel [113], 2013	Mice with targeted deletion of PiT-1 in VSMCs	Phosphate	High phosphate induced calcification of VSMCs.
El-Abbadi [115], 2009	Female DBA/2 mice induced uremia with left total nephrectomy	Phosphate	High phosphate was associated with extensive arterial medial calcification.
Graciolli [117], 2009	5/6 nephrectomized Wistar rats with parathyroidectomy	Phosphate	Phosphate upregulated aortic expression of Runx2 and led to calcified VSMC.
Hosaka [118], 2009	5/6 nephrectomized male Sprague-Dawley rats	Phosphate	High phosphate induced elastin degradation via the upregulation of tissue-nonspecific alkaline phosphatase, accelerating the transformation of VSMCs into osteoblast-like cells and leading to medial layer calcification.
Zhu [122], 2021	25 nephrectomized SPF-grade male Sprague–Dawley rats	Urea	Urea caused myocardial hypertrophy.
Prommer [124], 2018	11 uremic mice and 8 controls	Urea	Urea led to systemic microvascular disease, with microvascular rarefaction, tissue hypoxia, and dysfunctional angiogenesis.
Carmona [125], 2011	2 groups of 30 Wistar male rats: 1 with renal ablation and the other with kidney manipulation only	Urea	Urea induced systemic inflammation and led to the thickening of subepicardiac arteries.
			**Other than ATH and non ATH CVDs**
Yang [127], 2017	C57BL/6J mice with left total nephrectomy	IS	IS activated ROS/p38 MAPK signaling and reduced Klotho expression, which induced platelet aggregation and thrombus formation.
Kolachalama [128], 2018	A group of C57BL/6 mice administered Kyn, the excretion of which was inhibited by probenecid	Kynurenine	High kynurenine levels promoted clotting in response to vascular injury.
Koppe [133], 2017	5/6 nephrectomized	PCS	PCS (but not PCG) promoted insulin resistance.
	mice	PCG	
Koppe [134], 2013	CD1 Swiss and C57BL/6J mice with 5/6 nephrectomy	PCS	PCS contributed to insulin resistance: It altered insulin signaling in skeletal muscle through the activation of extracellular signal-regulated kinases.
Nagy [97], 2017	Male CD1 mice injected with CMPF	CMPF	CMPF inhibited insulin secretion.
Koppe [130], 2016	C57BL/6N male mice with 5/6 nephrectomy	Urea	Urea increased oxidative stress and protein O-GlcNAcylation, impairing insulin secretion and glycolysis.
Carracedo [131], 2013	5/6 nephrectomized 40 male Wistar rats	Urea	Urea induced oxidative stress, leading to EC damage.
D’Apolito [98], 2010	5/6 nephrectomized C57BL/6J wild-type mice	Urea	Urea increased ROS production and induced insulin resistance and glucose intolerance.
Li [132], 2018	5/6 nephrectomized rats	TMAO	High TMAO levels decreased NO production, contributing to endothelial dysfunction.
Zhu [129], 2016	Carotid artery thrombosis models of germ-free C57BL/6J female mice	TMAO	TMAO enhanced submaximal stimulus-dependent platelet activation, increasing the thrombosis risk.

Abbreviations: CMPF: 3-carboxy-4-methyl-5-propyl-2-furanpropanoic acid; CVD: cardiovascular disease; eNOS:endothelial nitric oxide synthase; HA: hippuric acid; IS:indoxyl sulfate; KA: kynurenic acid; MAPK: mitogen-activated protein kinase; MI: myocardial infarction; NADPH: nicotinamide adenine dinucleotide phosphate; NO: nitric oxide; PAD: peripheral artery disease; PCS: para-cresyl sulfate; PCG: p-cresyl glucuronide; ROS: reactive oxygen species; Runx2: runt-related transcription factor 2; TMAO: trimethylamine-N-oxide; UT: uremic toxin; VSMC: vascular smooth muscle cell.

### 3.3. Observational Studies

Observational studies of the UTs’ impact on ATH and/or non-ATH CVDs are summarized in Table 3.

Some of the studies sought to elucidate the association between UTs with ATH and/or non-ATH CVDs in patients with CKD.

In 147 patients with stage 1 to 5 CKD, high plasma IS levels were associated with major adverse cardiovascular events independently of the GFR and nutritional status [135]. A recent study by Konje et al. [136] found a positive association between kynurenine and incident ATH and non-ATH CVDs, including MI, angina, coronary artery bypass grafting, angioplasty/stenting of a coronary artery, stroke, PAD, congestive heart failure, and arrhythmia. Wu et al. [137] showed that elevated free PCS serum levels were associated with cardiovascular mortality in HD patients. A study by Liabeuf et al. [138] demonstrated for the first time that serum PCG levels were positively correlated with cardiovascular mortality, independently of survival predictors. These results showed that even though PCG is the minor metabolite of p-cresol, it appears to have a substantial impact on mortality—as much as PCS and IS do. Several studies confirmed the link between phosphate and CVDs and mortality. In 13,292 stage 3 to 5 CKD patients, serum phosphate was positively correlated with increased risk of incident stroke, transient ischaemic attack, MI, advanced coronary artery disease, new cardiac failure, and death [139]. An elevated serum phosphate concentration was significantly associated with cardiovascular mortality in many study settings [140,141,142,143]. Similarly, high serum TMAO levels were associated with cardiovascular events and death in HD patients, including coronary events, arrhythmias, sudden cardiac death, and congestive heart failure [144].

Urea has direct and indirect effects on adverse cardiovascular outcomes in patients with CKD. A recent cohort conducted by Laville et al. in 2022 [27] was the first to show a direct association between elevated serum urea concentrations and a greater risk of ATH CVD, non-ATH CVD, and mortality in pre-ESRD patients; this was independent of other conventional risk factors, including renal function. In addition, elevated urea levels were positively correlated with CVD. Nevertheless, this correlation was indirect. There are various hypotheses for the toxicity of high urea levels, including protein carbamylation (a post-translational modification of proteins with various biological consequences—mainly related to atherogenesis) [145]. Berg et al. [146] affirmed that high urea was positively correlated with the carbamylation of serum albumin, and suggested that this carbamylation had an impact on CVD and mortality.

All the above-mentioned previous studies concluded that there was a significant, independent association between UTs and the risk of cardiovascular complications. However, a few studies did not find an association after adjusting for confounders, and others did not find any association between some UTs and cardiovascular morbidity/mortality. The Chronic Renal Insufficiency Cohort (CRIC) study by Chen et al. [147] included 3407 patients with CKD but not ESRD. The results showed that lower 24-h kidney clearance of IS, KA, and PCS was associated with incident heart failure and MI; however, this association was not statistically significant after adjustment for the GFR. In the HEMO study, Shafi et al. concluded [148] that IS, PCS, PAG, and HA were not associated with any cardiovascular event (coronary events, peripheral vascular disease, ischemic heart disease, congestive heart failure, or arrhythmias). However, high IS levels were predictive of cardiac and sudden cardiac death in patients with lower albumin. It has been shown that high serum CMPF levels were not associated with any type of CVD [149], and that higher phosphate levels were associated with increased cardiovascular mortality; however, the latter association was not statistically significant after adjustment for GFR [150]. In a study of 235 HD patients, Kaysen et al. [151] found that there was no significant association between TMAO and cardiovascular hospitalizations or death.

Even though some studies investigated the association of UTs with both ATH and non-ATH CVDs, many focused on one or the other.

#### 3.3.1. Atheromatous Cardiovascular Diseases

The in vitro and in vivo effect of tryptophan-derived indoles on PAD (observed by Arinze et al. [50]) was confirmed in two cohorts. Firstly, a study of 20 HDs concluded that elevated plasma levels of IS, kynurenine, and KA were associated with a significant decrease in EC proliferation and migration, relative to 15 controls [50]. Secondly, 28 PAD patients with adverse limb events and 35 PAD patients without adverse limb events were followed up for 2 years. High plasma levels of IS, kynurenine, and KA, along with suppressed Wnt activity in ECs, were associated with an elevated risk of future adverse limb events [50]. In another study of 100 HD patients, elevated serum levels of IS and PCS were associated with PAD and arteriosclerosis markers [152].

Shafi et al. [153] concluded that higher serum levels of IS, PCS, PAG, and HA were associated with a greater risk of fatal or nonfatal atherosclerotic cardiovascular events. Previous studies had already shown that higher serum IS levels were associated with atherosclerotic cardiovascular events [154,155,156]. Hsu et al. [154] showed that higher serum IS levels were associated with coronary atherosclerosis and indicated that this elevation was correlated with the severity of the disease. In a cohort of 224 HD patients, plasma IS levels showed a significant negative correlation with HDL cholesterol, and they were associated with atherosclerotic lesions [156]. An older study by Lin et al. [157] in 2010 showed that high serum levels of PCS were significantly associated with atherosclerotic cardiovascular events. In 2016, Poesen et al. carried out two ancillary analyses of data from the Leuven Mild-to-Moderate CKD Study. One ancillary analysis showed that a lower serum PCS:PCG ratio and high total PCS and PCG levels were associated with fatal and nonfatal atherosclerotic CVDs [158]. The other demonstrated that an elevated serum PAG level was associated with CVD even after adjustment for age, sex, the presence of DM, prior CVD, and GFR; hence, elevated PAG was considered to be a powerful, independent risk factor for major CVDs (notably MI and stroke) [159].

The toxic effect of PCS on ATH CVDs dates back to a study conducted in 2010 on 202 stable angina patients with early stage of CKD, presenting that an elevated plasma level of PCS was associated with CAD and it was correlated with the severity of the disease [160]. The association between hyperphosphatemia and atherosclerotic diseases was reported from 2002 onwards [161] and again recently [162]. In 1203 non-dialyzed CKD patients, high serum phosphate levels were associated with an increased risk of fatal ATH CVD [163]. Hyperphosphatemia was associated with MI in a representative study of 3490 patients with CKD [164]. TMAO is the UT most frequently studied in animal models with regard to its link to ATH CVDs. Many cohorts from 2016 onwards have confirmed these findings. High TMAO concentrations were positively correlated with coronary atherosclerosis in one study [165] and with ischemic cardiovascular events in a study of 2529 stage 3b or 4 patients with CKD [166].

Only one study (by Melamed et al. [167]) failed to find an association between IS or PCS and fatal ATH CVD.

#### 3.3.2. Non-Atheromatous Cardiovascular Diseases

While TMAO is most studied compound with regard to the effect of UTs on ATH CVDs in vivo, its counterpart for non-ATH CVDs is IS. Most observational clinical studies have confirmed the association between IS and vascular calcification. A recent study of IS, IAA, PCS, PCG, and HA showed that each UT was significantly and negatively correlated with peak cardiac power and significantly and positively correlated with subclinical cardiac dysfunction but not with the left ventricular mass index [168]. Cao et al. [169] and Shimazu et al. [170] reported that high plasma IS was associated with heart failure [169,170] and cardiac death [170]. In a study of 204 patients with CKD and preserved left ventricular function, greater plasma IS levels were associated with an elevated risk of left ventricular diastolic dysfunction [171]. In a cohort study by Barreto et al. [172], serum IS levels were directly associated with pulse wave velocity and aortic calcification, and being in the highest IS tertile was a strong predictor of cardiovascular mortality. In a study in 2020, KA also was associated with diastolic dysfunction [173]. Serum KA levels were positively correlated with aortic stiffness and with indices of left atrium and left ventricle diastolic dysfunction [173]. In addition, high plasma kynurenine and KA levels were associated with intima-media thickness in 106 CKD patients [174]. A 2010 study by Liabeuf et al. [175] proved that an elevated serum total and free PCS levels were significantly associated with vascular calcification, and free PCS was shown to be a predictor of cardiovascular mortality. HA was also investigated in 2018; high HA levels were significantly associated with left ventricular hypertrophy in HD patients [176]. Several studies showed that hyperphosphatemia was associated with vascular calcification [177,178,179]. Petchey et al. [177] further reported that serum phosphate was positively correlated with the aortic pulse wave velocity and thus with arterial stiffness. In a cohort of 6814 patients with CKD, high serum phosphate was associated with vascular and valvular calcification [178]. Moreover, Ix et al. [180] concluded that hyperphosphatemia was strongly associated with peripheral arterial stiffness. As mentioned above, urea exerts its toxicity indirectly through protein carbamylation. In 1255 HD patients, greater blood urea levels were associated with serum carbamylated albumin levels, and being in the upper urea tertile was positively correlated with heart failure and arrhythmia [181].

#### 3.3.3. Other than Atheromatous and Non-Atheromatous Cardiovascular Diseases

Many observational studies have highlighted the detrimental effect of UTs on ECs. A novel study by Glorieux et al. [182] in 2021 demonstrated that high serum levels of IS, IAA, PCS, PCG, and HA were correlated with markers of endothelial damage—mainly angiopoietin-2, a protein with an essential role in angiogenesis and that is involved in carotid artery intima media thicknening, arterial stiffness, and left ventricular hypertrophy [182,183]. In a 2019 study by Wang et al. [184], elevated serum IS levels were negatively correlated with vascular reactivity index values. A study by Jourde-Chiche et al. [185] found that high serum levels of IS, IAA, and PCS were associated with low numbers of endothelial progenitor cells. Meijers et al. [66] investigated PCS in vitro and in an observational study; high serum PCS levels were associated with the presence of circulating endothelial microparticles. In 2009 and 2010, Pawlak et al. studied the toxicity of kynurenine and its metabolite KA; both were shown to be associated with elevated oxidative stress, inflammation, and EC dysfunction [186]. The researchers found that plasma KA levels were associated positively with TF inhibitor levels and negatively with prothrombin fragment 1 + 2 levels [187]. Pawlak et al. also concluded that plasma kynurenine levels were positively associated with thrombomodulin and von Willebrand factor (both markers of EC dysfunction) [188].

In addition, high serum kynurenine and KA levels were independently and significantly associated with hypercoagulability [189]. Elevated serum IS and kynurenine levels contribute to postangioplasty complications. High serum levels of IS [128,190] and kynurenine [128] were associated with postangioplasty thrombosis of dialysis grafts.

It is noteworthy that high levels of free PCS and free PCG had the strongest association with CVDs even after adjustment for the GFR [182].

**Table 3:** Observational studies of the effects of UTs on cardiovascular complications.

First Author, Year	Models	UT(s) Studied	Main Findings
			**Atheromatous CVDs**
Arinze [50], 2022	20 HD patients and 15	IS	Elevated plasma levels of IS, kynurenine, and KA in
	controls	Kynurenine	HD patients showed a significant decrease in ECs
		KA	proliferation and migration, compared with the control group.
Arinze [50], 2022	PAD patients: 35 without	IS	Elevated plasma levels of IS, kynurenine, KA, with
	adverse limb event and	Kynurenine	suppressed Wnt activity in ECs were associated with
	28 with	KA	an increased risk of future adverse limb events.
Shafi et al. [153], 2015	394 incident HD patients	IS	Elevated serum levels of IS, PCS, PAG and HA were
		PCS	associated with greater risk of fatal or nonfatal
		PAG	atherosclerotic cardiovascular events in incident HD
		HA	patients.
Hsu [154], 2013	191 mild-to-moderate CKD patients	IS	Elevated serum IS levels were associated with coronary atherosclerosis and correlated with the severity of the disease.
Melamed [167], 2013	521 incident HD patients	IS	IS and PCS were not associated with atherosclerotic
		PCS	cardiovascular death.
Lin [155], 2012	70 pre-dialysis patients (CKD stage 3 to 5)	IS	Serum IS levels were positively correlated with atherosclerotic cardiovascular events.
Lin [152], 2012	100 stable HD patients	IS	Elevated serum levels of IS and PCS were associated
		PCS	with PAD and arteriosclerosis markers.
Lin [157], 2010	100 HD patients	IS	Only elevated serum PCS levels were significantly
		PCS	associated with fatal or nonfatal atherosclerotic cardiovascular events.
Taki [156], 2007	224 HD patients	IS	Plasma IS levels were significantly and negatively correlated with HDL cholesterol and were positively associated with atherosclerotic lesions.
Poesen [158], 2016	488 patients (all CKD	PCS	A lower serum PCS:PCG ratio and a higher
	stages)	PCG	total PCS + PCG level were associated with fatal or nonfatal atherosclerotic CVDs.
Wang [160], 2010	202 patients with stable angina and early-stage kidney failure	PCS	Elevated plasma PCS levels were associated with coronary artery disease and correlated with the severity of the disease.
Poesen [159], 2016	488 patients with CKD stages 1–5	PAG	An elevated serum PAG level was a powerful, independent risk factor for major CVD (such as MI and stroke).
Merhi [162], 2017	3138 CKD patients	Phosphate	Hyperphosphatemia was associated with atherosclerotic CVD.
Eddington [163], 2010	1203 nondialyzed CKD patients	Phosphate	Hyperphosphatemia increased the risk of cardiovascular death from atheromatous CVD.
Kestenbaum [164], 2005	3490 CKD patients	Phosphate	Hyperphosphatemia was associated with MI.
Nakamura [161], 2002	525 HD patients	Phosphate	Hyperphosphatemia was associated with atherosclerotic diseases.
Stubbs [165], 2016	104 CKD patients	TMAO	Elevated TMAO concentrations were correlated with coronary atherosclerosis.
Kim [166], 2016	2529 patients (stages 3b and 4 CKD)	TMAO	Elevated serum TMAO levels were associated with ischemic cardiovascular events.
			**Non-atheromatous CVDs**
Chinnappa [168], 2018	56 male patients with	IS	These serum UT levels showed significant negative
	stage 2–5 CKD, nondia-	IAA	correlation with peak cardiac power and subclinical
	lyzed and free of heart	PCS	cardiac dysfunction, but no correlation with left ven-
	disease	PCG	tricular mass index was found.
		HA	
Cao [169], 2015	258 HD patients	IS	Elevated plasma IS was associated with heart failure.
Sato [171], 2013	204 CKD patients with preserved left ventricular function	IS	Elevated plasma IS levels were associated with an increased risk of left ventricular diastolic dysfunction.
Shimazu [170], 2013	76 patients with mild-to-moderate CKD and dilated cardiomyopathy	IS	Elevated serum IS levels were associated with hospitalization for heart failure and cardiac death.
Barreto [172], 2009	139 patients with CKD from stage 2 to dialysis	IS	Being in the highest serum IS tertile was directly associated with pulse wave velocity, aortic calcification, and higher cardiovascular mortality.
Zapolski [173], 2020	100 CKD patients with persistent atrial fibrillation	KA	Serum KA levels were positively correlated with aortic stiffness and indices of diastolic dysfunction of left atrium and left ventricle.
Pawlak [174], 2010	106 CKD patients	KA	Elevated plasma kynurenine and KA levels were as-
		Kynurenine	sociated with intima-media thickness.
Liabeuf [175], 2010	139 CKD patients	PCS	Elevated total and free serum PCS levels were significantly associated with vascular calcification, and free PCS was shown to be a predictor of cardiovascular death.
Yu [176], 2018	80 HD patients	HA	Elevated HA levels were significantly associated with left ventricular hypertrophy.
Petchey [177], 2012	120 CKD pre-dialysis patients	Phosphate	Serum phosphate was positively correlated with aortic pulse wave velocity, arterial stiffness, and the presence of vascular calcification.
Adeney [178], 2009	6814 patients with CKD aged 45–84	Phosphate	Hyperphosphatemia was associated with vascular and valvular calcification.
Ix [180], 2009	440 patients with moderate CKD	Phosphate	Hyperphosphatemia was strongly associated with peripheral arterial stiffness.
Ketteler [179], 2003	312 HD patients	Phosphate	Hyperphosphatemia was associated with vascular calcification and cardiovascular mortality.
Drechsler [181], 2015	1255 HD patients	Urea	Higher blood urea levels were associated with higher tertile serum carbamylated albumin levels, which in turn were positively correlated with heart failure and arrhythmia.
			**Atheromatous and non-atheromatous CVDs**
Chen [147], 2021	3407 participants with	IS	Lower 24-h kidney clearance of IS, KA, and PCS
	CKD, excluding those	KA	was not found to be associated with heart failure and
	with a GFR < 20 mL/min/1.73 m^2^	PCS	MI after adjustment for GFR.
Fan [135], 2019	147 patients with CKD stage 1–5	IS	Elevated plasma IS levels were associated with major adverse cardiovascular events, independently of GFR and nutritional status.
Shafi [148], 2017	1273 HD patients	IS	Overall, elevated serum IS, PCS, PAG and HA levels
		PCS	were not associated with any cardiovascular event.
		PAG	However, high IS levels were predictive of cardiac
		HA	and sudden cardiac death in patients with low albumin levels.
Konje [136], 2021	92 CKD patients with a history of CVD, 46 with no history of CVD, and 46 with incident CVD	Kynurenine	Elevated serum kynurenine levels were associated with incident atheromatous and non-atheromatous CVDs.
Wu [137], 2012	112 HD patients aged from 65 to 90	PCS	Elevated free PCS serum levels were associated with cardiovascular mortality.
Liabeuf [138], 2013	139 CKD patients	PCG	Elevated free and total serum PCG levels were correlated with cardiovascular mortality independently of survival predictors.
Luce [149], 2018	270 HD patients	CMPF	Elevated serum CMPF was not associated with any CVD.
McGovern [139], 2013	13,292 CKD patients at stages 3–5	Phosphate	Hyperphosphatemia was correlated with increased CVDs.
Kimata [140], 2007	3973 HD patients	Phosphate	Hyperphosphatemia was significantly associated with cardiovascular mortality.
Menon [150], 2005	840 CKD patients	Phosphate	Hyperphosphatemia was significantly associated with increased cardiovascular mortality but only before adjustment for GFR.
Slinin [141], 2005	14829 HD patients	Phosphate	Hyperphosphatemia was associated with CVDs and mortality.
Young [142], 2005	17236 dialysis patients	Phosphate	Hyperphosphatemia was significantly associated with cardiovascular mortality.
Block [143], 2004	40538 HD patients	Phosphate	Hyperphosphatemia was significantly associated with cardiovascular hospitalization and mortality.
Laville [27], 2022	2507 CKD patients before RRT	Urea	Higher serum urea levels were associated with a greater risk of CVD.
Berg [146], 2013	187 HD patients	Urea	Urea was positively correlated with carbamylation of serum albumin, which is associated with CVDs and mortality.
Shafi [144], 2017	1846 prevalent HD patients	TMAO	An elevated serum TMAO concentration was associated with cardiovascular events and death.
Kaysen [151], 2015	235 HD patients	TMAO	There was no significant association between TMAO and cardiovascular hospitalizations or death.
			**Other than ATH and non ATH CVDs**
Glorieux [182], 2021	523 nondialyzed patients	IS	Elevated serum levels of these UTs were correlated
	(all stages of CKD)	IAA	with markers of endothelial damage (mainly angio-
		PCS	poietin-2). Elevated levels of free PCS and free PCG
		PCG	had the strongest association with CVD, indepen-
		HA	dently of the GFR.
Wang [184], 2019	110 patients with stage 3–5 CKD	IS	Elevated levels of serum IS were negatively correlated with vascular reactivity index values, leading to endothelial dysfunction.
Kolachalama [128], 2018	473 participants under-	IS	Elevated serum levels of IS and kynurenine were
	going angioplasty for dialysis access dysfunction	Kynurenine	associated with postangioplasty thrombosis of dialysis grafts.
Wu [190], 2016	306 patients undergoing angioplasty for dialysis access dysfunction	IS	Elevated serum levels of IS were associated with postangioplasty thrombosis of dialysis grafts.
Jourde-Chiche [185], 2009	38 HD patients and 21	IS	Elevated serum levels of IS, IAA, and PCS were asso-
	healthy controls	IAA	ciated with low numbers of endothelial progenitor
		PCS	cells.
Pawlak [187], 2010	64 patients on peritoneal dialysis	KA	Plasma KA levels were positively associated with TF inhibitor and negatively associated with prothrombin fragment 1 + 2 levels.
Pawlak [188], 2009	48 patients with ESRD	Kynurenine	Plasma kynurenine levels were positively associated with thrombomodulin and von Willebrand factor (markers of endothelial dysfunction).
Pawlak [186], 2009	146 CKD patients with 91	Kynurenine	Elevated serum levels of kynurenine and KA were
	ones on dialysis	KA	associated with increased oxidative stress, inflammation, and endothelial dysfunction.
Pawlak [189], 2009	92 patients on dialysis	Kynurenine	Elevated serum levels of kynurenine and KA were
		KA	independently and significantly associated with hypercoagulability.
Meijers [66], 2009	100 HD patients	PCS	Elevated serum PCS levels were associated with the levels of circulating endothelial microparticles.

Abbreviations: CMPF: 3-carboxy-4-methyl-5-propyl-2-furanpropanoic acid; CKD: chronic kidney disease; CVD: cardiovascular disease; EC: endothelial cell; ESRD: end-stage renal disease; GFR: glomerular filtration rate; HA: hippuric acid; HD: hemodialysis; HDL: high-density lipoprotein; IAA: indole-3-acetic acid; IS:indoxyl sulfate; KA: kynurenic acid; MI: myocardial infarction; PAD: peripheral artery disease; PAG: phenylacetylglutamine; PCS: para-cresyl sulfate; PCG: p-cresyl glucuronide; RRT: renal replacement therapy; TF: tissue factor; TMAO: trimethylamine-N-oxide; UT: uremic toxin.

## 4. Conclusions

We primarily reviewed gut-derived UTs because most of the latter are protein-bound solutes that are difficult to remove from the plasma; their accumulation in the circulation is associated with many harmful effects (including cardiovascular complications). We also reviewed phosphate and urea, whose toxicity has long been known but is still being explored. CVD is a major problem in patients with CKD and constitutes the leading cause of death in this population [19]. Strategies implemented to modify UT levels have been described in previous reviews [11,191]. A combined approach of targeting UTs with the other aspects of CVD could be necessary for its prevention. Noteworthy, the relation between CKD and CVDs is bidirectional: CVDs could also impact kidney’s health, causing kidney damage and disease progression, creating the cardio-renal syndrome [192].

## Data Availability

Not applicable.
